# P-2124. Retrospective Study to Determine the Predictive Factors for Invasive Fungal Infections in Patients who had undergone Bone Marrow Transplant

**DOI:** 10.1093/ofid/ofaf695.2288

**Published:** 2026-01-11

**Authors:** Haritha Subhagan, Dipu T Sathyapalan, Kiran G kulirankal, Neeraj Sidharthan, Nikhil Krishna Haridas, J S Gayathri, Jahnavi Mohandas, Georg Gutjahr, Merlin Moni

**Affiliations:** Amrita Institute of Medical Sciences, Kochi, Kerala, India; Professor, Department of Internal Medicine, Lead Division of Infectious diseases, Administrative chair, URUM, chairman HICC, Kochi, Kerala, India; Amrita Institute of Medical Sciences and Research Center, kochi, Kerala, India; Amrita Institute of Medical Sciences, Kochi, Kerala, India; Amrita Institute of Medical Sciences, Kochi, Kerala, India; Amrita Institute of Medical Sciences, Kochi, Kerala, India; Amrita Institute of Medical Sciences, Kochi, Kerala, India; Amrita Vishwa Vidyapeetham University, Kochi, Kerala, India; Amrita Institute of Medical Sciences, Kochi, Kochi, Kerala, India

## Abstract

**Background:**

Hematopoietic stem cell transplant (HSCT) recipients face a heightened risk for invasive fungal Infections (IFIs), which are a major cause of morbidity and mortality. Due to limited data from India, we investigated the prevalence and risk factors of IFIs following HSCT.

The Sankey plot illustrates the treatment journey and the outcome of the patients who has undergone HSCT.

**Figure:**
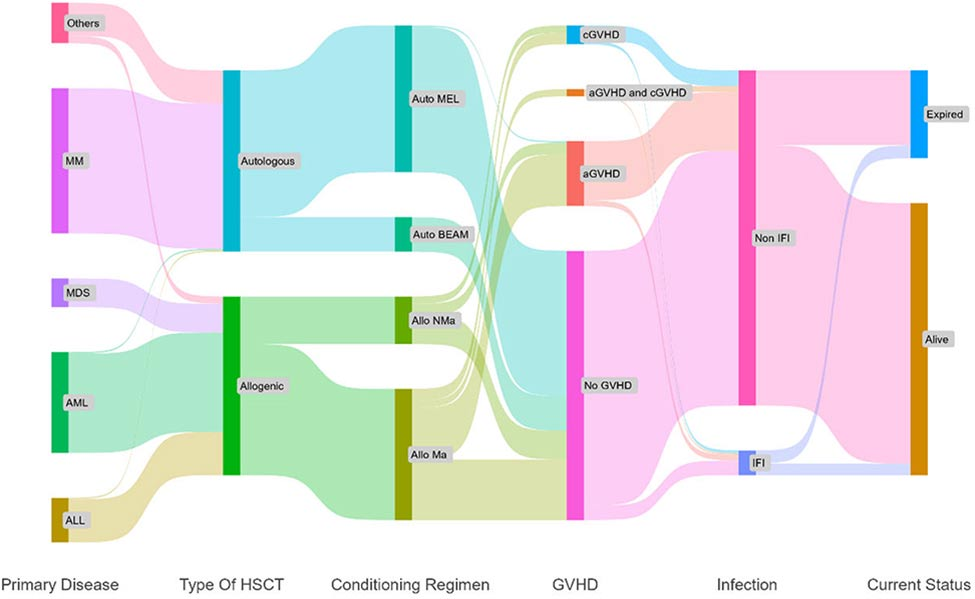

It flows from left to right. Each colored stem represents a group of patients, and the width of each stream indicates the number of patients at each stage. Abbreviations: MM, Multiple Myloma; MDS, Myelodysplastic Syndrome; AML, Acute Myeloid Leukemia; ALL, Acute Lymphoblastic Leukemia; Auto MEL, Autologous transplant with Melphalan; Auto BEAM, Autologous transplant with BEAM protocol; Allo NMa, Allogenic transplant with non-myeloablative conditioning; Allo Ma, Allogenic transplant with myeloablative conditioning; IFI, Invasive Fungal Infection.

**Methods:**

This single-centre retrospective study from South India included all HSCT recipients from 2013 to 2023. Medical records of a sample were reviewed for risk factors and outcomes. IFI cases were defined as per 2020 EORTC/MSG criteria. Multivariate logistic regression was used to identify independent IFI predictors. A Sankey plot illustrated treatment pathways and outcomes post-HSCT

**Results:**

The incidence of IFIs after HSCT was 6.8% (17/250). The rate of IFIs was notably higher among those with Myelo Dysplastic Syndrome (MDS) comprising 35% (6/17), followed by AML 24% (4/17) and others. Allogenic transplant recipients had a greater IFI rate of 12% (15/124) when compared to autologous recipients 1.5% (2/126). Invasive Aspergillosis was the predominant IFI 64.7% (11/17), followed by invasive candidiasis 29.4% (5/17) and Mucormycosis 5.9% (1/17). Primary disease, type of HSCT, donor type, conditioning regimen, ANC engraftment and CMV reactivation were the significant predictors / risk factors for developing IFI. Antifungal prophylaxis given was Fluconazole 54% (134/250), Micafungin 38% (94/250), posaconazole 5.6% (14/250) and voriconazole 3.2% (8/250). Majority (54.55%) of the patients in the MDS group who received Micafungin (antiyeast prophylaxis) developed IFI. The Sankey plot which illustrates the distribution of patients who had undergone HSCT based on their primary diseases, type of HSCT, conditioning regimen, graft versus host disease (GVHD), infection status and clinical outcome is shown in figure. The 28days all-cause mortality was 12% in the IFI group and 3.4% in the non IFI group. Overall survival and non-relapse mortality were significantly worse in IFI patients (*p* = 0.007 and *p* = 0.008 respectively).

**Conclusion:**

This retrospective study highlights the need to optimise antifungal prophylaxis, especially in MDS patients. It emphasizes enhanced surveillance and targeted prophylaxis in allogeneic transplant recipients with identified risk factors.

**Disclosures:**

All Authors: No reported disclosures

